# The Batten disease gene Cln3 is required for the activation of intestinal stem cell during regeneration via JAK/STAT signaling in *Drosophila*

**DOI:** 10.3389/fcell.2025.1508714

**Published:** 2025-01-23

**Authors:** Zihua Yu, Jinhua Yan, Zhiming Liu, Haiyan Wang, Guanzheng Luo, Haiyang Chen

**Affiliations:** ^1^ MOE Key Laboratory of Gene Function and Regulation, Guangdong Province Key Laboratory of Pharmaceutical Functional Genes, State Key Laboratory of Biocontrol, School of Life Sciences, Sun Yat-sen University, Guangzhou, China; ^2^ West China Centre of Excellence for Pancreatitis and Laboratory of Stem Cell and Anti-Aging Research, National Clinical Research Center for Geriatrics, West China Hospital, Sichuan University, Chengdu, Sichuan, China

**Keywords:** *Drosophila*, intestinal stem cell (ISC), Batten disease, CLN3, JAK/STAT signaling pathway

## Abstract

CLN3 mutation causes Juvenile neuronal ceroid lipofuscinosis (JNCL, also known as Batten disease), an early onset neurodegenerative disorder. Patients who suffer from Batten disease often die at an early age. However, the mechanisms underlying how CLN3 loss develops Batten disease remain largely unclear. Here, using *Drosophila* midgut system, we demonstrate that *Drosophila* Cln3 has no effect on midgut homeostasis maintaince, including cellular component, intestinal stem cells (ISCs) proliferation and differentiation, but is necessary for ISC activation upon tissue damage. Cell type-specific Gal4 screening reveals that the failure of ISC activation during regeneration caused by Cln3 loss is ISC-autonomous. Through genetic analyses, we elucidate that JAK/STAT signaling in ISCs is not activated with Cln3 depletion upon tissue damage, and functions downstream of Cln3. Our study provides a potential mechanism underlying the development of CLN3-mediated Batten disease at cellular level.

## 1 Introduction

Juvenile neuronal ceroid lipofuscinosis (JNCL), also known as Batten disease, is a neurodegenerative disorder that affects young individuals (Lerner et al., 1995). Afflicted children display progressive vision loss without any precursor symptoms. Within a span of 2–4 years, patients experience personality changes, behavioral problems, and cognitive decline, followed by the loss of motor functions, ultimately leading to premature death, often before the age of 25 (Nita et al., 2016). The NCL diseases are characterized by the accumulation of ceroid lipofuscin within lysosomes. One suggested consequence of this accumulation is the disruption of vital cellular processes, resulting in cell death and subsequent neurodegeneration (Cooper, 2003). However, the precise mechanisms underlying Batten disease at the cellular level, particularly in stem cells, remain largely unknown.

Researches have identified mutated genes relevant to Batten disease named ceroid-lipofuscinosis, neuronal (*CLN*) 1-14 (Nita et al., 2016). Among them, *CLN3* is responsible for JNCL and is the most common type of NCLs. The gene *CLN3* has been found to be highly conserved across yeast, fruit fly, mouse and human. The coding product, CLN3 protein, is highly hydrophobic, with six transmembrane helices and is predicted to be constitutively expressed (Mirza et al., 2019). Studies have confirmed its subcellular localization within the membrane of lysosomes or endosomes (Kyttala et al., 2004). Numerous investigations have suggested that CLN3 is involved in various cellular functions, including endocytosis (Fossale et al., 2004; Metcalf et al., 2008), lysosomal homeostasis (Chattopadhyay et al., 2000; Gachet et al., 2005), mitochondrial function (Fossale et al., 2004; Luiro et al., 2006) and autophagy (Cao et al., 2006). Our previous research has demonstrated the requirement of CLN3 in the response to oxidative stress (Tuxworth et al., 2011). However, the functions of CLN3 in adult stem cells still remain unexplored.

Adult stem cells play a crucial role in maintaining tissue and organ homeostasis by continuously replacing functional cells (Post and Clevers, 2019). When tissues experience injury or loss of functional cells, there is a requirement for adult stem cells to rapidly proliferate and differentiate in order to replenish the damaged areas. The adult *Drosophila* digestive tract has been established as a useful system for the study of adult stem cell proliferation due to the advantages of simple cellular component and feasible genetic manipulation (Nászai et al., 2015). Similar to the mammalian intestine, the *Drosophila* midgut epithelium undergoes constant replenishment through intestinal stem cells (ISCs) (Ohlstein and Spradling, 2005; Micchelli and Perrimon, 2005). Upon activation, a quiescent ISC can undergo symmetric division to expand the population of stem cells, or asymmetric division to generate a new ISC and a committed progenitor cell called an enteroblast (EB), which subsequently differentiates into a mature polyploid enterocyte (EC) (Ohlstein and Spradling, 2007). On rare occasions, an ISC can undergo asymmetric division and produce a distinct type of progenitor cell known as an enteroendocrine mother cell (EMC). The EMC then goes through symmetric division, resulting in the formation of a pair of enteroendocrine (EE) cells that contribute to the maintenance of homeostasis (Guo and Ohlstein, 2015). To date, no research on the role of CLN3 in ISCs has been published.

In this study, we aimed to investigate the impact of Cln3 loss in *Drosophila* intestinal stem cells. Under conditions of homeostasis, the loss of Cln3 does not affect ISCs. However, when the midgut is exposed to stress, Cln3 loss results in the failure of ISC activation. This phenotype is cell-autonomous and caused by the lack of activating of the JAK/STAT pathway in ISCs. These results shed light on a novel role of Cln3 in adult stem cells and provide a potential mechanism underlying Batten disease.

## 2 Results

### 2.1 Cln3 does not affect midgut homeostasis under normal condition in *Drosophila*

**FIGURE 1:**
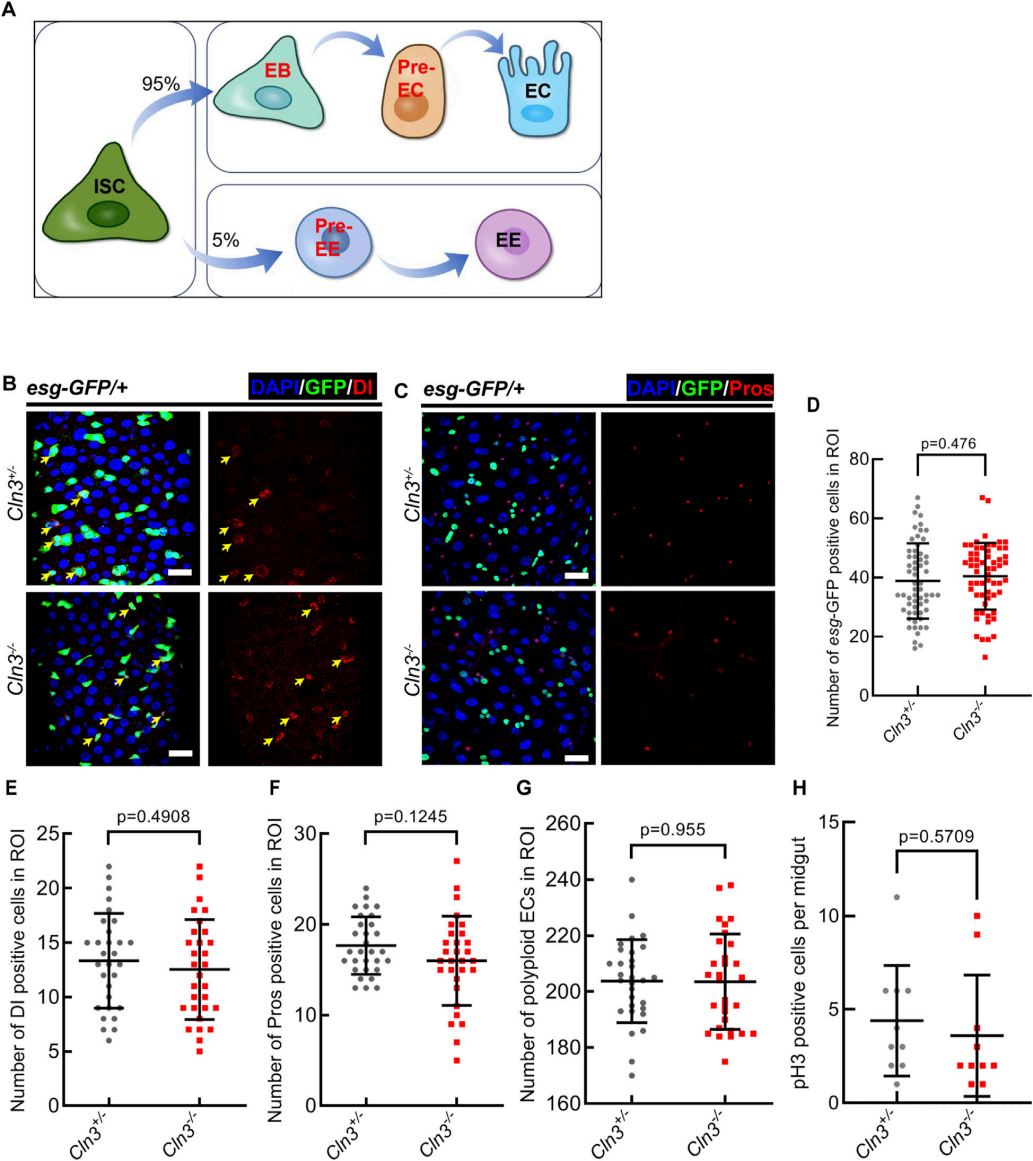
Cln3 is not necessary in *Drosophila* midgut homeostatsis maintainence. **(A)** A model of cell lineage in *Drosophila* midgut epithelium. **(B)** Representative immunofluorescence of midguts with *esg*-GFP (green) and Dl (red) staining from control (*Cln3* ^*+/−*^) flies and Cln3 mutant (*Cln3* ^*−/−*^) flies. Yellow arrows indicate Dl^+^ ISCs. **(C)** Representative immunofluorescence of midguts with *esg*-GFP (green) and Pros (red) staining from control (*Cln3* ^*+/−*^) flies and Cln3 mutant (*Cln3* ^*−/−*^) flies. **(D–G)** Quantification of the number of *esg* ^*+*^ cells **(D)**, Dl^+^ cells **(E)**, Pros^+^ cells **(F)** and polyploid ECs **(G)** per region of interest (ROI) of midguts in experiments **(B, C)**. ROI size 84,100 μm^2^. **(H)** Quantification of the number of pH3^+^ cells per midgut in experiments **(B, C)**. DAPI-stained nuclei (blue). Scale bar, 25 μm. Bars are mean ± SD. Statistics were measured by two-tailed, unpaired Student’s t-test. n = 60 and 60 ROIs from 30 midguts each in **(D)**, n = 30 and 30 ROIs from 15 midguts each in **(E–G)**, n = 10 and 10 midguts in **(H)**, respectively. Each experiment was repeated for 3 times.

To explore the function of Cln3 in *Drosophila* midgut, we previously generated a Cln3 mutant line named *Cln3* ^*ΔMB1*^ by imprecise excision of a transposable element (Tuxworth et al., 2011). This allele exhibites a deletion of 1,534 bp and can be viewed as a null (Tuxworth et al., 2011). This homozygote mutant survived to adulthood and showed no phenotype in the midgut epithelium (a model of cell lineage was shown in Figure 1A) from the following aspects: (Lerner et al., 1995): *escargot* ^+^ (*esg* ^+^, a promotor activating in stem and progenitor cells) cells (Figures 1B–D); (Nita et al., 2016) Delta^+^ (Dl^+^, the ligand of Notch signaling) intestinal stem cells (ISCs) (Figures 1B, E); (Cooper, 2003) Prospero^+^ (Pros) enteroendocrine cells (EEs) (Figures 1C, F); (Mirza et al., 2019) polyploid enterocytes (ECs) (Figures 1B, C, G). Also, phosphorylated histone H3 (pH3, marks mitotic cells) staining revealed no difference in ISC proliferation between *Cln3* ^*ΔMB1*^ mutant and wild type flies (Figure 1H). These data suggested that *Cln3* is not a necessary gene under homeostasis condition in the *Drosophila* midgut.

### 2.2 Cln3 is required for the activation of ISCs upon tissue damage in *Drosophila*

**FIGURE 2:**
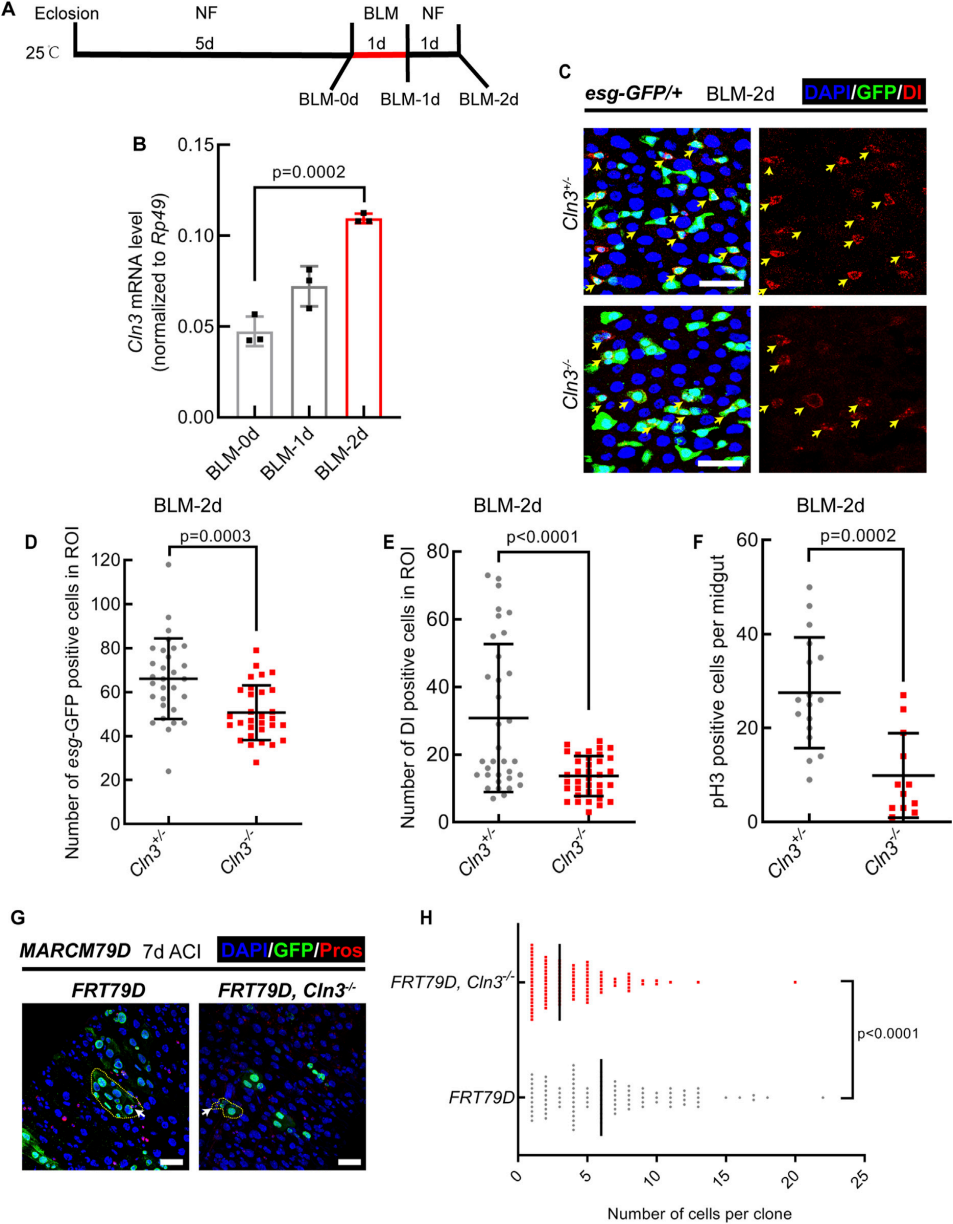
Cln3 activates ISC proliferation during regeneration. **(A)** Diagram of the experimental procedure of fly treatment. **(B)** The relative mRNA expression level of Cln3 from whole midguts of wild type flies (*w* ^*1118*^) at indicated time points. **(C)** Representative immunofluorescence of midguts with *esg*-GFP (green) and Dl (red) staining from control (*Cln3* ^*+/−*^) flies and Cln3 mutant (*Cln3* ^*−/−*^) flies at the time point of BLM-2d. Yellow arrows indicate Dl^+^ ISCs. **(D, E)** Quantification of the number of *esg* ^*+*^ cells **(D)** and Dl^+^ cells **(E)** per ROI of midguts in experiment **(C)**. ROI size 84,100 μm^2^. **(F)** Quantification of the number of pH3^+^ cells per midgut in experiment **(C)**. **(G)** Immunofluorescence analysis of clones induced by *MARCM79D* of control (*FRT79D*) and Cln3 mutant (*FRT79D, Cln3* ^*−/−*^). Pros (red) staining was used to indicate EEs. Yellow dotted lines indicated the border of clones. White arrows indicated Pros^+^ EEs inside the clones. **(H)** Quantification of the number of cells per clone in experiment **(G)**. DAPI-stained nuclei (blue). Scale bar, 25 μm. Bars are mean ± SD. Statistics were measured by one-way ANOVA in **(B)** and two-tailed, unpaired Student’s t-test in **(D, E)** and **(H)**. n = 30 and 30 ROIs from 15 midguts each in **(D)**, n = 34 and 36 ROIs from 17 to 18 midguts in **(E)**, n = 17 and 12 midguts in **(F)**, n = 100 and 100 clones from 20 midguts each in **(H)**, respectively. Each experiment was repeated for 3 times.

Since we previously demonstrated that *Cln3* ^*ΔMB1*^ mutant flies showed no phenotype in normal condition but were hypersensitive to oxidative stress (Tuxworth et al., 2011), we asked whether Cln3 also functions only under stress condition in the midgut. To test this, we fed *Drosophila* with bleomycin (Figure 2A) and *Erwinia carototovovora carototovovora* 15 (*Ecc15*) (Supplementary Figure S1), which induced DNA break of ECs and infection of the midgut, respectively, and subsequent activation of ISCs to replenish the damage of epithelium (Guo et al., 2013). We first tested the RNA expression level of Cln3 in the midgut and found it upregulated after bleomycin and *Ecc15* treatment (Figure 2B; Supplementary Figure S1A). This suggested that Cln3 may play a role in the regeneration process. Then, we checked the changes of ISC behavior. As a result, while the percentage of *esg* ^+^ and Dl^+^ cells increased in wild type flies after bleomycin and *Ecc15* treatment, *Cln3* ^*ΔMB1*^ mutant failed to exhibit this increase (Figures 2C–E; Supplementary Figures S1B–D). Moreover, pH3 staining revealed fewer proliferative stem cells in the *Cln3* ^*ΔMB1*^ mutant (Figure 2F; Supplementary Figure S1E). These results indicated that Cln3 plays a role in the proliferation of ISCs during regeneration. Since both of bleomycin and *Ecc15* treatment revealed similar results, we used only bleomycin for further investigation.

To further support this, we used mosaic analysis with a repressible cell marker (MARCM) system (Lee and Luo, 1999), in which only active ISCs are able to generate a clone. Results revealed that compared to wild type clones, *Cln3* ^*ΔMB1*^ mutant clones were smaller (Figures 2G, H), suggesting weaker mitotic ability of mutated ISCs. Noticed that both wild type clones and mutant clones contained Pros^+^ EEs (Figure 2G), which further supported the conclusion that Cln3 did not affect ISC differentiation.

### 2.3 ISCs are responsible for their activation during regeneration upon Cln3 loss

**FIGURE 3:**
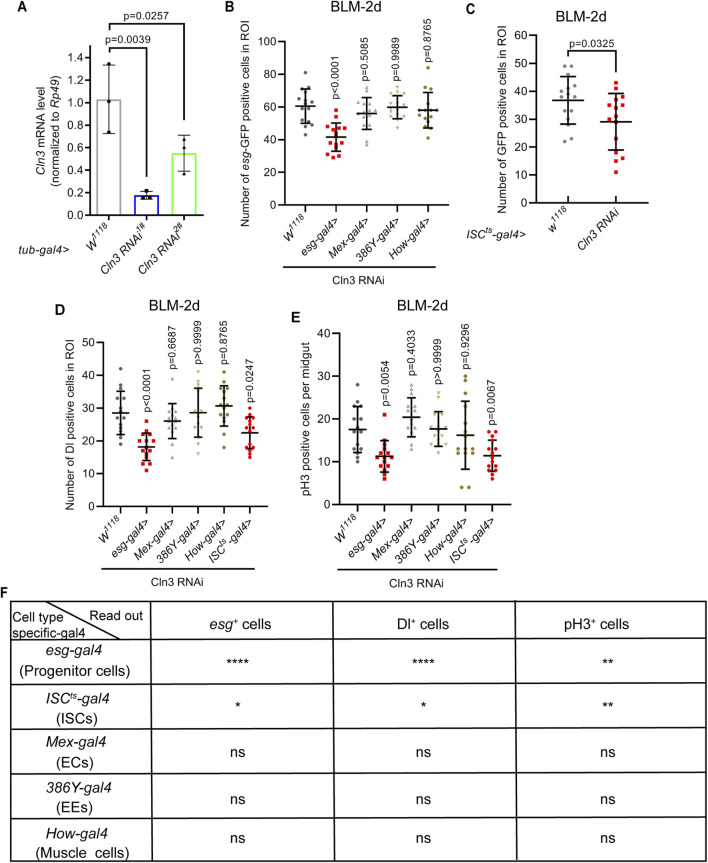
Gal4 screening reveals ISCs responsible for their activation upon Cln3 loss during regenaration. **(A)** The relative mRNA expression level of Cln3 from whole flies of control (*tub-gal4*, *w* ^*1118*^) and Cln3 depletion (*tub-gal4>Cln3 RNAi* ^*1#*^, *tub-gal4>Cln3 RNAi* ^*2#*^). Each experiment was repeated for 3 times. **(B–D)** Quantification of the number of *esg-*GFP^*+*^ cells **(B)**, *ISC* ^*ts*^ *-gal4*>GFP^+^ cells (indicate *esg* ^+^ cells without *NRE* expression) **(C)** and Dl^+^ cells **(D)** per ROI of midguts with indicated genotypes. ROI size 84,100 μm^2^. **(E)** Quantification of the number of pH3^+^ cells per midgut with indicated genotypes. **(F)** Summary of the results of gal4 screening. Only *esg-gal4* and *ISC* ^*ts*^ *-gal4* driven Cln3 RNAi led to significant differences. *, p < 0.05; **, p < 0.01; ****, p < 0.0001; ns, not significant. Statistics were measured by one-way ANOVA in **(A, B)** and **(D, E)**, and two-tailed, unpaired Student’s t-test in **(C)**. n = 15 ROIs from 8 midguts each in all groups of **(B–D)**, and n = 15 midguts in all groups of **(E)**. Each experiment was repeated for 3 times.

Next, we sought to figure out which cell type is responsible for this failure of ISC activation. We first tested the efficiency of two Cln3 RNAi lines. Results revealed that both of these two RNAi lines driven by whole body expressed *tub-gal4* exhibited over 60% of Cln3 mRNA depletion (Figure 3A), suggesting high RNAi efficiency. Then, we used different kinds of cell type specific-gal4 to drive Cln3 RNAi (stem and progenitor cell-specific *esg-gal4*; ISC-specific *esg-gal4, NRE-gal80, tub-gal80* ^*ts*^ (shortened as *ISC* ^*ts*^ *-gal4*); EE-specific *386Y-gal4*; EC-specific *Mex-gal4* and muscle-specific *How-gal4*). These results combinely suggested that the failure of ISC activation during regeneration was cell-autonomous, because only *esg-gal4* and *ISC* ^*ts*^ *-gal4* driven Cln3 RNAi led to the failure of the increase of *esg* ^+^ cells, Dl^+^ cells and pH3^+^ cells upon bleomycin treatment (Figures 3B–F).

### 2.4 Cln3 activates ISC proliferation during regeneration through JAK/STAT signaling pathway

**FIGURE 4:**
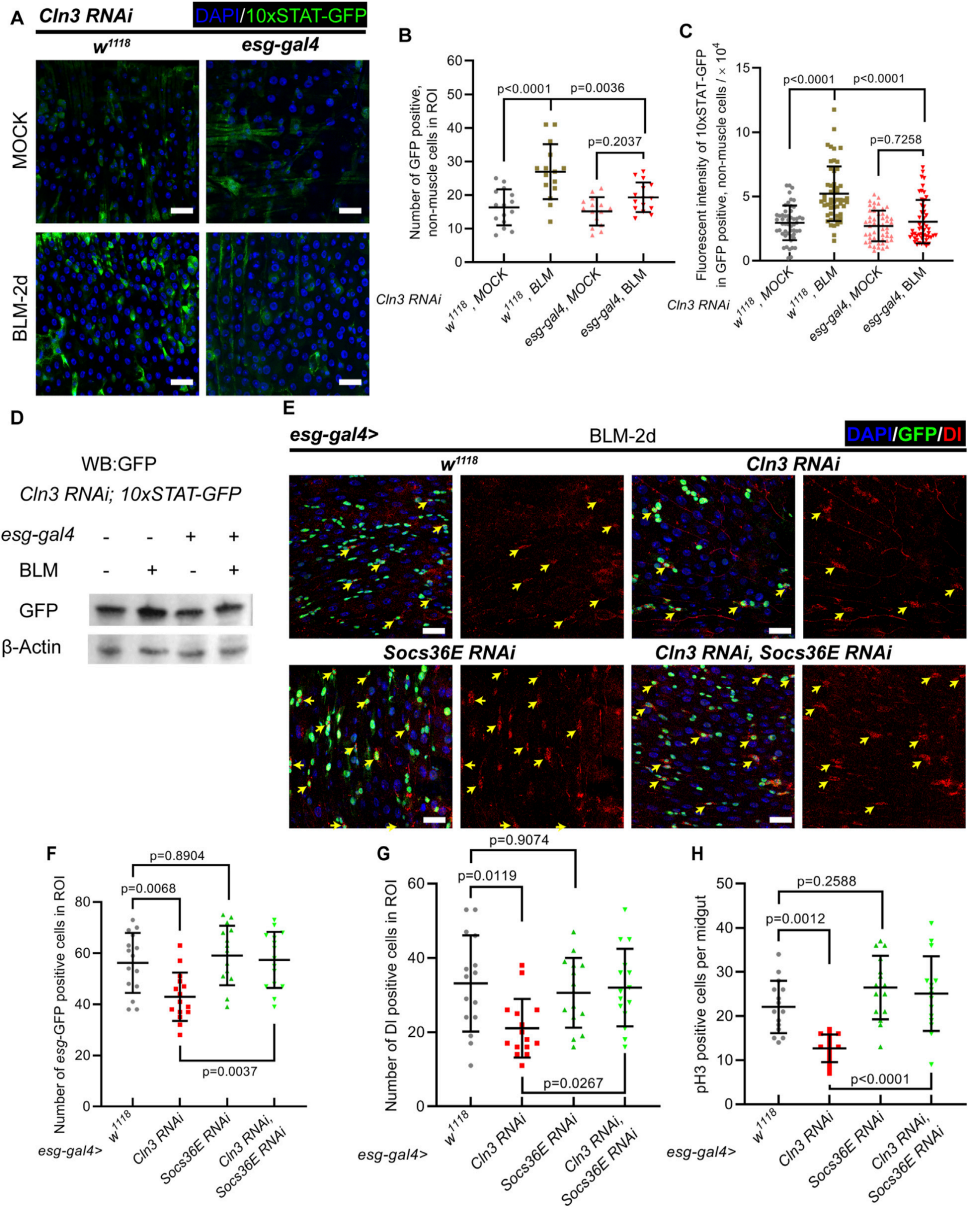
JAK/STAT signaling functions downstream of Cln3 to activate ISCs during regeneration. **(A)** Representative immunofluorescence images of midguts from control (*w* ^*1118*^ *, Cln3 RNAi, 10 × STAT-GFP*) and Cln3 depletion (*esg-gal4>Cln3 RNAi, 10 × STAT-GFP*) flies with MOCK (sucrose) or BLM treatment. 10 × STAT-GFP staining indicated the activity of JAK/STAT signaling pathway. **(B, C)** Quantification of the number of 10 × STAT-GFP^*+*^, non-muscle cells per ROI **(B)** and fluorescent intensity of 10 × STAT-GFP in 10 × STAT-GFP^*+*^, non-muscle cells **(C)**. **(D)** Western blot analysis of 10 × STAT-GFP protein level from whole midgut in experiment **(A)**. **(E)** Representative immunofluorescence of midguts with *esg*-GFP (green) and Dl (red) staining from control (*esg-gal4*, *w* ^*1118*^), or flies expressing *UAS-Cln3 RNAi* or *UAS-Socs36E RNAi* single depletion, and *UAS-Cln3 RNAi, UAS-Socs36E RNAi* double depletion driven by *esg-gal4.* Yellow arrows indicate Dl^+^ ISCs. **(F, G)** Quantification of the number of *esg* ^*+*^ cells **(F)** and Dl^+^ cells **(G)** per ROI of midguts in experiment **(H)**. ROI size 84,100 μm^2^. **(H)** Quantification of the number of pH3^+^ cells per midgut in experiment **(E)**. DAPI-stained nuclei (blue). Scale bar, 25 μm. Bars are mean ± SD. Statistics were measured by one-way ANOVA. n = 15 ROIs from 8 midguts each in all groups of **(B)** and **(F, G)**; n = 53, 52, 50 and 50 cells from 8 midguts each in **(C)**, respectively and n = 15 midguts in all groups of **(H)**. Each experiment was repeated for 3 times.

Then, we asked the mechanism underlying how Cln3 regulates ISC proliferation during regeneration. JAK/STAT signaling is well reported to regulate ISC proliferation (Jiang et al., 2009; Zhai et al., 2017; Osman et al., 2012). During regeneration, ECs that undergo apoptosis secreate the ligand of JAK/STAT signaling, Unpaired 3, which binds to the receptor Domeless expressed through the plasma membrane of ISCs, to activate ISC proliferation (Osman et al., 2012). To test whether Cln3 depletion has an impact on JAK/STAT signaling in ISCs, 10 × STAT-GFP reporter line was used (Bach et al., 2007). During regeneration, while the control group showed more cells with 10 × STAT-GFP positive signal, stronger 10 × STAT-GFP signal per cell, and higher GFP protein level, these changes were not significant in the Cln3 depletion group (Figures 4A–D). Moreover, we tested the mRNA level of two STAT-target genes, Socs36E and Dpp, which revealed similar results (Supplementary Figure S2A). These data suggested that JAK-STAT signaling was not able to switch on with Cln3 depletion.

To further verify the function of JAK/STAT signaling upon Cln3 depletion, we tested the genetic relationship between JAK/STAT signaling and Cln3. Apart from the function of reporting JAK/STAT signaling, Socs36E is also a vital negative regulator which mediates degradation of positive regulator STAT92E (Issigonis et al., 2009). We drove Socs36E RNAi to upregulate JAK/STAT signaling. During regeneration, while wild type flies exhibited increased *esg* ^+^ cells, Dl^+^ cells and pH3^+^ cells, these numbers reached a lower level in Cln3 depletion group (Figures 4E–H). In the Cln3 and Socs36E double depletion group, the *esg* ^+^ cell number, Dl^+^ cell number and pH3^+^ cell number showed the same level to Socs36E depletion group (Figures 4E–H). These data indicated that JAK/STAT signaling pathway functioned downstream of Cln3 in response to stress.

## 3 Discussion

JNCL is an uncurable inherited metabolic disorder caused by *CLN3* mutation. It is meaningful to uncover more details about the disease. To date, the function of *CLN3* is not clearly demonstrated (Mirza et al., 2019). Particularly, the mechanisms underlying how CLN3 loss develops the disease remains largely unknown. Using the *Drosophila* midgut system as a model, here we demonstrate that *Drosophila* Cln3 is required for the activation of ISCs during regeneration. Upon damage of midgut, ISCs of Cln3 mutant fail to undergo fast proliferation to replenish the loss of functional cells. Genetic analysis reveals that this failure is ISC-autonomous through JAK/STAT signaling pathway (Supplementary Figure S3).

We have previously reported that in *Drosophila*, Cln3 mutant exhibits hypersensitive to oxidative stress (Tuxworth et al., 2011). In this study, we uncover that Cln3 mutant fails to activate ISCs upon tissue damage. These two findings suggest that in *Drosophila*, Cln3 is not necessary for homeostasis maintainance, but becomes vital under stress condition. To validate this, further investigations should focus on the survival performance and stem cell behavior of Cln3 mutant under various stress conditions, though we have proved some (Tuxworth et al., 2011). In particular, whether adult stem cells are able to mediate regeneration promptly, is urgent to be demonstrated. If this applies to more types of stem cells and more kinds of stresses, it will safely comes to the conclusion that Cln3 is required for adult stem cell activation during regeneration. This will further shed light on the mechanisms underlying how CLN3 mutation develops JNCL in human. It is possible that CLN3 mutation limits adult stem cells to produce new progenies after cell death caused by accumulation of ceroid lipofuscin, which subsequently results in more severe organ degeneration.

We have previously demonstrated the behavior of ISCs during regeneration in the *Drosophila* midgut (Du et al., 2020). In wild type flies, the proliferation rate of ISCs first increases sharply, and gradually slows down to the level before exposed to stress. The time point BLM-2d in this study shows the highest ISC proliferation rate in the regeneration model (Du et al., 2020), which sidely supports the role of Cln3 in the *Drosophila* midgut. Failure of the activation of ISCs to proliferate results in the longer existence of injury, as well as a shortened lifespan. This may also contribute, at least in part, to the development of Batten disease.

Specifically, our previous work has revealed that RAB7-mediated endocytosis functions upstream of JAK/STAT signaling pathway to regulate ISC regeneration (Du et al., 2020). Combined with the hint that CLN3 was reported to be involved in endocytosis (Fossale et al., 2004; Metcalf et al., 2008), we tried to find the direct relationship between Cln3 and RAB7 by generating a Cln3-HA-knock in line. However, while DNA sequence revealed by PCR showed correct knock in, the HA reporter cannot be detected even through western blot analysis. This may suggest complicated epigenetic regulation of the Cln3 locus. Therefore, the direct mechanisms underlying how CLN3 regulates JAK/STAT signaling and subsequent phenotype of Batten disease should be investigated by other methods or in other organisms.

The role of JAK/STAT signaling in the development of Batten disease still remains largly unknown. Based on the reported functions of JAK/STAT signaling pathway, 4 possible mechanisms could be put forward: (Lerner et al., 1995): Inflammation and immune response. Batten disease is associated with chronic inflammation in the brain. The JAK/STAT pathway plays a crucial role in regulating immune responses and inflammatory processes. Dysregulation of this pathway may contribute to the immune dysfunction observed in Batten disease, leading to increased inflammation and subsequent neuronal damage (Nita et al., 2016). Neuroprotection and cell survival. Activation of the JAK/STAT pathway has been shown to promote cell survival and protect against apoptosis. In Batten disease, the progressive loss of neurons contributes to the symptoms. Dysfunctional JAK/STAT signaling could impair neuroprotection mechanisms, making neurons more susceptible to degeneration (Cooper, 2003). Neuronal development and maturation. The JAK/STAT pathway is involved in neurodevelopmental processes such as neuronal proliferation, differentiation, and migration. A disruption in the JAK/STAT pathway in Batten disease may result in impaired neuronal development and maturation, contributing to the progressive neurodegeneration observed in affected individuals (Mirza et al., 2019). Oxidative stress and antioxidant defense. Batten disease is associated with increased oxidative stress, which can lead to cellular damage. The JAK/STAT pathway has been implicated in regulating antioxidant defense mechanisms, which help counteract oxidative stress. Dysregulation of this pathway may compromise the antioxidant defense system, further aggravating oxidative damage in Batten disease.

Nevertheless, limitations about studying Batten disease using *Drosophila* model are obvious. The most important is that the Cln3 mutant *Drosophila* does not develop any JNCL-like phenotypes, nor accumulates intracellular autofluorescent materials (Tuxworth et al., 2011). Although CLN3 is structurally conservd from yeast to human, its functions, particularly in material transport through lysosome, may have changed during evolution. Therefore, more details about the functions of CLN3 relevant to lysosome should be investigated in other organisms.

## 4 Methods

### 4.1 *Drosophila* stocks

The fly stocks used in this study are listed as follows: wild type line: *w* ^*1118*^ *;;* (BDSC: 3605); Cln3 mutant line: *;;Cln3* ^*ΔMB1*^ (Our lab); *esg*-GFP reporter line: *;esg-GFP/CyO;* (From Allan Spradling); 10 × STAT-GFP reporter line: *;10 × STAT-GFP;* (From Zheng Guo); *tub-gal4* line: *;;tub-gal4/TM3.e.Sb* (From Allan Spradling); *esg-gal4* line: *;esg-gal4/CyO;* (From Allan Spradling); *ISC* ^*ts*^ *-gal4* line: *Su(H)-lacZ;esg-gal4.UAS-GFP/Kr-GFP.CyO;tub-gal80ts.GBE-gal80/MKRS* (From Zheng Guo); *Mex-gal4* line: *;Mex-gal4;* (From Zheng Guo); *386Y-gal4* line: w[*];; P{w[+mW.hs] = GawB}386Y (BDSC: 25410); *How-gal4* line: w[*];; P{w[+mW.hs] = GawB}how[24B] (BDSC: 1767); Cln3 RNAi 1# line: TH201500324.S; Cln3 RNAi 2# line: THU1691; Socs36E RNAi line: THU1666; UAS-GFP reporter line: *w[1118];; P{w[+mC] = UAS-GFP.nls}8* (BDSC: 4776). FRT79D line: *;;FRT79D* (From Zheng Guo); MARCM79D tool line: *yw.hsflp.tub-gal4::GFP/FM7;;FRT79D, tub-gal80/TM6B* (From Zheng Guo).

### 4.2 *Drosophila* husbandry

All flies were cultured at 25°C at a 12 h light/dark cycle. For the Gal4/Gal80^ts^ system, the offspring were shifted to 29°C 5 days after eclosion for removing Gal80 inhibition and enabling Gal4 to drive *UAS*-linked transgene expression for another 5 days. For MARCM system, flies were raised at 25°C until 5 days after eclosion. Then the flies were transferred to a new vial and incubated in 37°C water bath for 1-h-heat shock. Flies were transferred back to 25°C for another 7 days for clone growth followed by dissection.

### 4.3 Bleomycin and *Ecc15* treatment

*Drosophila* were transferred from the medium to empty bottles for 2 h. The filter paper was cut into 3.5 × 6.0 cm pieces and treated with 5% (wt/vol) sucrose with 25 μg/mL bleomycin (BLM). Then, the moist papers were added to empty bottles for 24 h. BLM-1d group flies were dissected at this point, and the rest flies were transferred into a new standard medium without BLM for another 24 h before dissection. For Ecc15 infection, an infection solution was prepared by mixing an equal volume of 100× concentrated pellet from an overnight culture of Ecc15 (OD_600_ = 200) with a solution of 5% sucrose. One piece of filter paper treated with this infection solution was placed into a bottle with standard food. After treatment with empty bottles for 2 h, *Drosophila* were transferred to the bottles with standard food and filter paper for 24 h followed by dissection. Filter paper treated with 5% sucrose were used as control.

### 4.4 Immunofluorescence microscopy for *Drosophila* tissues

Adult fly intestines were dissected at 4°C in PBS and fixed with a mixture of 100 µL 4% EM-grade paraformaldehyde fixation buffer and 100 µL n-Heptane for 20 min. The intestines were washed in 200 µL methanol for 2 times, 5 min each. They were subsequently rinsed in 200 µL PBS plus 0.1% Triton X-100 (Sigma, PBST) for 2 times, 5 min each. The tissues were then incubated with primary antibodies dissolved in 0.1% PBST for overnight at 4°C. Then they were washed with 0.1% PBST for 3 times, 5 min each before secondary antibodies and DAPI (1:1,000; Sigma) incubated for 2 h at room temperature. After secondary antibodies incubation, the intestines were washed with 0.1% PBST for 3 times, 5 min each. The sources and dilutions of antibodies used are listed as follows: Chicken anti-GFP (1:1000; ab13970, Abcam), Rabbit anti-GFP (1:1000; 50430-2-AP, Proteintech), Prospero antibody (1:200; MR1A, DSHB), Delta antibody, (1:50; C594,9B, DSHB), and Alexa Fluor secondary antibodies (1:2000; A11004, A11008, A11011, A11039, A32733 and A32933). Leica TCS-SP8 confocal microscope was used to acquire all immunofluorescence images. For each set of experiments, images were acquired as confocal stacks using the same settings.

### 4.5 RNA isolation and RT-qPCR for *Drosophila*

50 adult midguts or 10 whole flies were collected into 4°C diethylpyrocarbonate (DEPC)-treated water-PBS solution. Samples were homogenized in RNA-easy Isolation Reagent (Vazyme, R701) for total RNA isolation and cDNA synthesis. RT-qPCR was performed on a CFX96 Touch Deep Well (Bio-Rad) using ChamQ Universal SYBR qPCR Master Mix (Vazyme, Q711). The reference standard group was Rp49. The expression levels were counted by the 2^−△△CT^ method. The primers used were listed as below:

Rp49-F: GCC​CAA​GGG​TAT​CGA​CAA​CA.

Rp49-R: GCG​CTT​GTT​CGA​TCC​GTA​AC.

CLN3-F: TCG​TCG​GGA​AGA​AAC​TGT​CAC.

CLN3-R: GAG​CTA​TAC​GGA​AAT​TCA​CCC​AA.

Socs36E-F: GCT​GCC​AGT​CAG​CAA​TAT​GT.

Socs36E-R: GAC​TGC​GGC​AGC​AAC​TGT.

Dpp-F: TGG​CGA​CTT​TTC​AAA​CGA​TTG​T.

Dpp-R: CAG​CGG​AAT​ATG​AGC​GGC​AA.

### 4.6 Fluorescence intensity statistics

Immunofluorescence imaging results were analyzed based on z-stacks acquired with confocal microscopy. The fluorescence intensity of the region of interest (ROI) or cell was calculated using ImageJ software following the fomular:

Integrated Density=Integrated Density of ROI or cell—Integrated Density of background region/Area of background region×Area of ROI or cell.

### 4.7 Western blotting

50 midguts were collected for protein extraction. The primary antibodies used for western blotting in this study were: anti-GFP (rabbit, 1:5000, 50430-2-AP, Proteintech); and anti-β-actin (rabbit, 1:2000, PA5-85271, Invitrogen). The secondary antibody was horseradish peroxidase-conjugated goat anti-rabbit (1:5000, Jackson ImmunoResearch Labs, Cat# 211-032-171).

## Data Availability

The original contributions presented in the study are included in the article/Supplementary Material, further inquiries can be directed to the corresponding author.

## Supplementary material

SUPPLEMENTARY FIGURE S1 Cln3 activates ISC proliferation upon infection. **(A)** The relative mRNA expression level of Cln3 from whole midguts of wild type flies (*w* ^*1118*^) with or without *Ecc15* treatment. **(B)** Representative immunofluorescence of midguts with *esg*-GFP (green) and Dl (red) staining from control (*Cln3* ^*+/-*^) flies and Cln3 mutant (*Cln3* ^*-/-*^) flies with or without *Ecc15* treatment. Yellow arrows indicate Dl^+^ ISCs. **(C, D)** Quantification of the number of *esg* ^*+*^ cells **(C)** and Dl^+^ cells **(D)** per ROI of midguts in experiment **(B)**. ROI size 84,100 μm^2^. **(E)** Quantification of the number of pH3^+^ cells per midgut in experiment **(B)**. DAPI-stained nuclei (blue). Scale bar, 25 μm. Bars are mean ± SD. Statistics were measured by two-tailed, unpaired Student’s *t*-test. n = 30 and 30 ROIs from 15 midguts each in **(C, D)** and n = 15 and 15 midguts in **(E)**, respectively. Each experiment was repeated for 3 times.

SUPPLEMENTARY FIGURE S2 The expression of STAT-target genes reveals regulation by Cln3. **(A)** The relative mRNA expression level of *Socs36E* and *Dpp* from whole midguts of flies with indicated genotype with or without BLM treatment. The expression level was normalized to *rp49*. The relative expression level of the MOCK groups was further normalized to 1 and marked as the dotted line. Bars are mean ± SD. Statistics were measured by one-way ANOVA. Each experiment was repeated for 3 times.

SUPPLEMENTARY FIGURE S3 Proposed model. Left: Cln3 loss does not affect homeostasis. Right: During regeneration, Cln3 loss represses the activation of ISCs through JAK/STAT signaling pathway, which may contribute to Battern disease.
