# Supplementary figures and images for: The Batten disease gene Cln3 is required for the activation of intestinal stem cell during regeneration via JAK/STAT signaling in Drosophila
Source: Front Cell Dev Biol. 2025 Jan 23;13:1508714. doi: 10.3389/fcell.2025.1508714 (PMC11799272; doi:10.3389/fcell.2025.1508714)

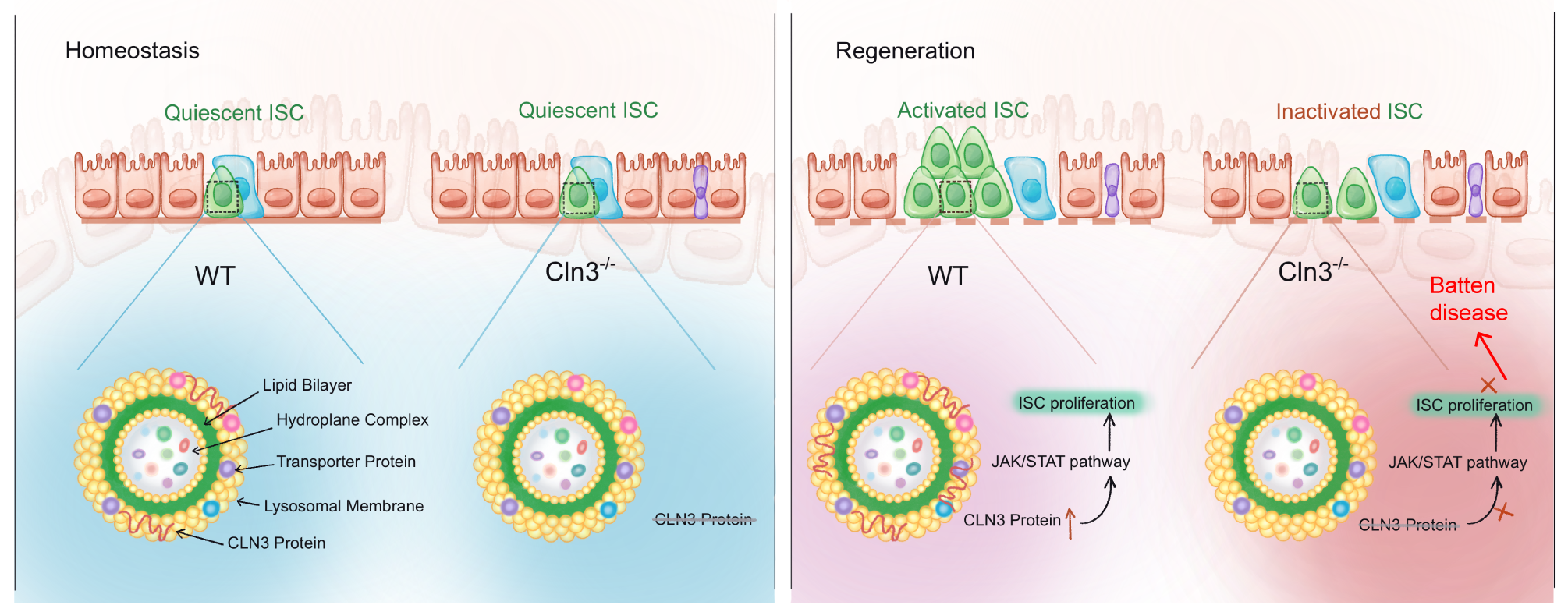

Supplement: Supplementary file 1 [file Image3.tif]

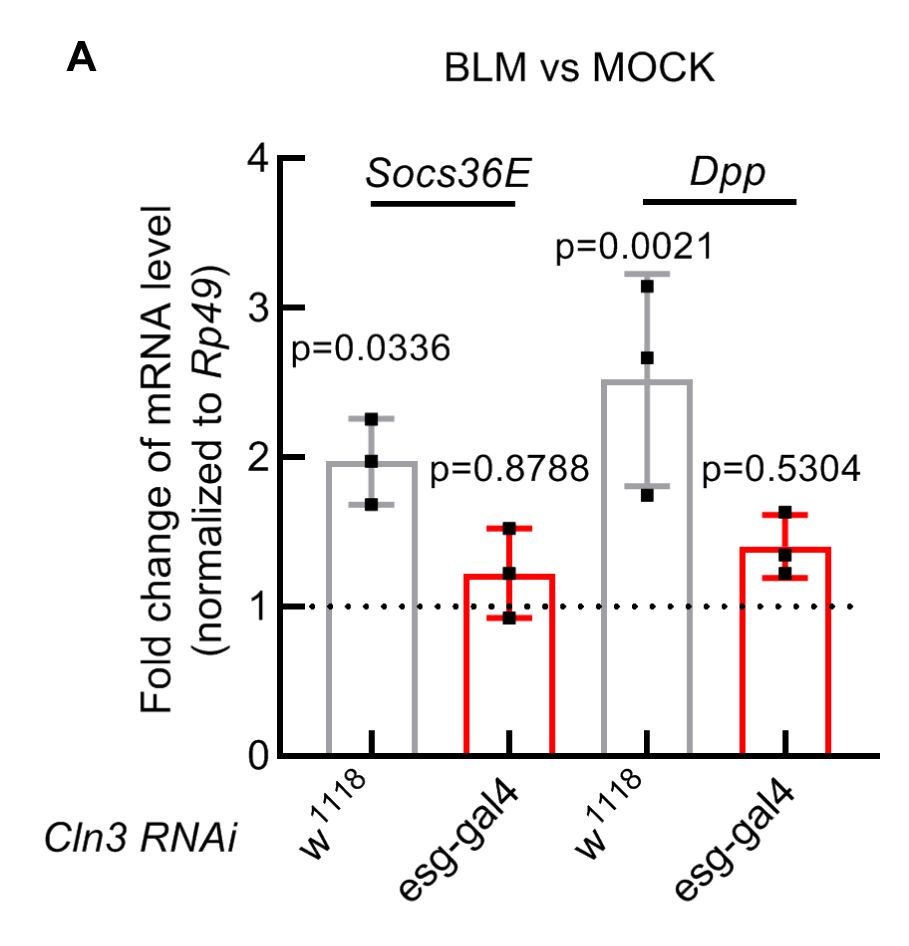

Supplement: Supplementary file 2 [file Image2.tif]

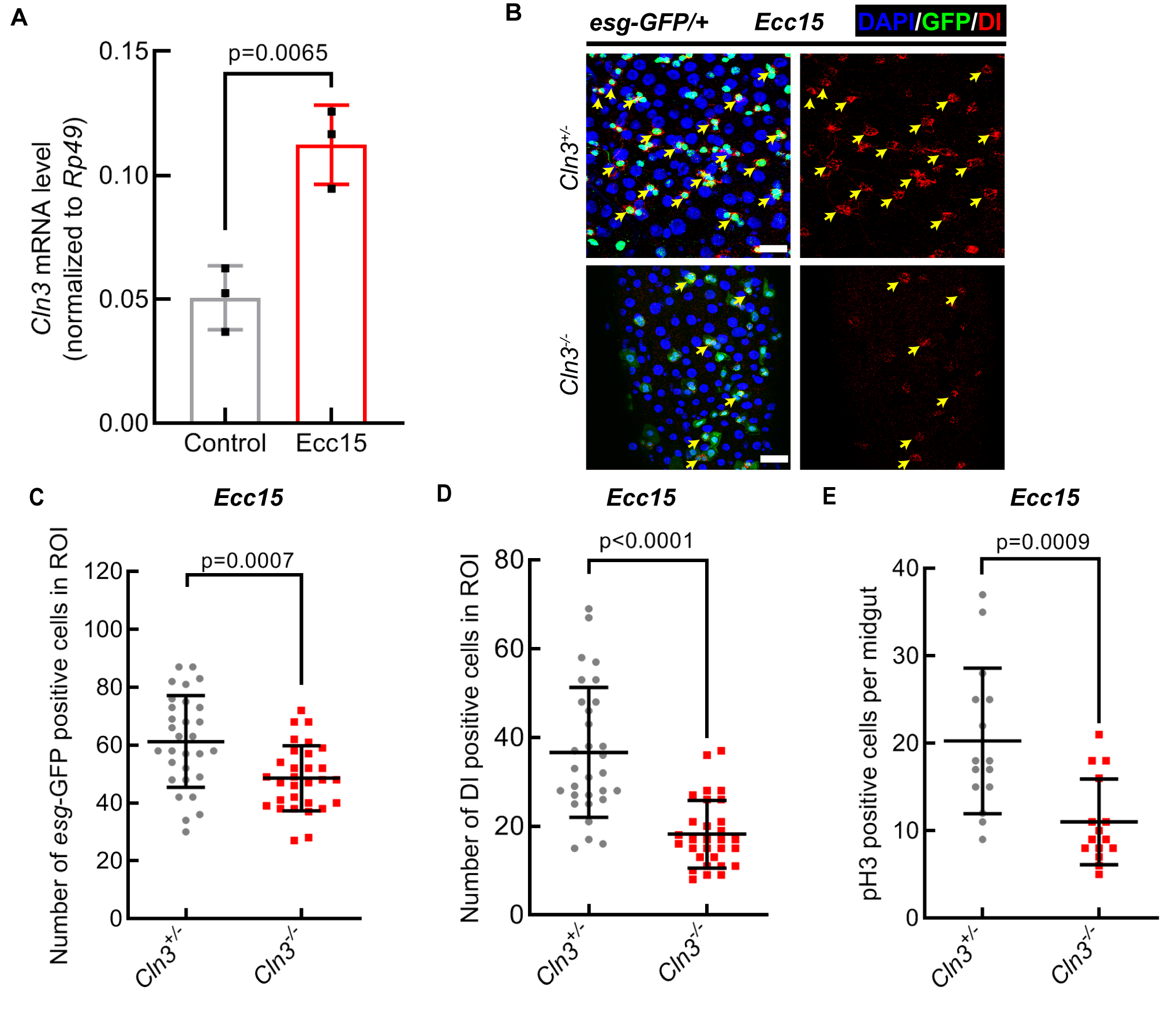

Supplement: Supplementary file 3 [file Image1.tif]
